# Menthol’s disruptive effects on kanamycin-resistant *Escherichia coli* energy metabolism and ion fluxes

**DOI:** 10.1016/j.bpr.2025.100240

**Published:** 2025-11-20

**Authors:** Silvard Tadevosyan, Siranuysh Grabska, Hovakim Grabski, Ruben Abagyan, Karen Trchounian, Naira Sahakyan

**Affiliations:** 1 Research Institute of Biology, Yerevan State University Department of Biochemistry, Microbiology & Biotechnology, Yerevan State University, Yerevan, Armenia; 2 L.A. Orbeli Institute of Physiology, National Academy of Sciences, Yerevan, Armenia; 3 Skaggs School of Pharmacy and Pharmaceutical Sciences, University of California, La Jolla, San Diego, CA 92093-0657

## Abstract

The presented study aimed to investigate the antibacterial activity of menthol—the main component of one of the widespread plants of the Lamiaceae family—*Mentha arvensis*. To investigate the mode of action of menthol, we studied its influence on kanamycin-resistant *E. coli* pARG-25 and wild-type *E. coli* BW25113 strains. For this, the effect of menthol on ATPase activity, proton and potassium fluxes, and intracellular pH was investigated under aerobic and anaerobic conditions. The results showed that menthol influences these parameters in a concentration- and condition-dependent way. It likely interacts with FoF_1_-ATPase and other systems involved in energy-generating processes and ion transport, disrupting the bacterial metabolism of both antibiotic-resistant and -susceptible strains.

## Why It Matters

The antibiotic resistance problem demands new antimicrobials with alternative mechanisms of action. This study reveals that menthol disrupts bacterial bioenergetics by affecting FoF_1_-ATPase activity, ion fluxes, and membrane potential in both antibiotic-susceptible and -resistant *Escherichia coli*. The effects were concentration and oxygen dependent, with stronger inhibition in the kanamycin-resistant strain. Bioinformatic analysis supported the interaction of menthol with multiple molecular targets, highlighting its role as a membrane-active agent. These findings provide mechanistic insight into menthol’s antibacterial activity and emphasize its potential as a natural modulator of energy metabolism in resistant bacteria, contributing to the development of new membrane-targeting antimicrobial strategies.

## Introduction

A wide range of metabolites derived from plants contribute to diverse aspects of plant growth and development in response to external factors. They serve crucial roles in both plant adaptation and survival while also holding significant value across various human activities (1,2,3,4,5,6).

The antimicrobial activity of plant-derived metabolites is among the most extensively investigated biological property, largely due to the growing global concern over antibiotic resistance, which poses a significant challenge to modern healthcare systems and imposes a substantial burden on clinical management and nursing practices (7,8,9,10,11,12,13,14). In recent decades, particular attention has been directed toward the antimicrobial activity of essential oils, especially those derived from aromatic and medicinal plants (13,15,16,17,18). Despite the large number of in vitro and in vivo results, the biochemical mechanisms underlying their antimicrobial efficacy remain incompletely understood.

Among the suggested mechanisms, the disruptive effects of plant-derived metabolites on bacterial membranes, compromising membrane integrity and leading to cell lysis, have reported (7,14,17,19).

The essential oil derived from *Mentha* genus species of Lamiaceae family is particularly rich in menthol, a monoterpenoid compound possessing antimicrobial activity against a vast range of microorganisms (20). Alongside menthone and isomenthone, menthol contributes to the characteristic minty aroma and cooling sensation of mint-derived oils. Before its isolation and structural elucidation, menthol was exclusively sourced from natural plant material. The compound exists in eight stereoisomeric forms, with (−)-menthol being the most prevalent isomer in both natural and synthetic sources (21).

Menthol is of high commercial demand, with applications spanning pharmaceuticals, cosmetics, food, and tobacco industries. According to recent market analyses, the global menthol market was valued at approximately $1,008.82 million in 2022 and is projected to reach $1,241.76 million by 2028, growing at a compound annual growth rate of 3.52% (MarketWatch, 2023). Its broad utility in diverse sectors underscores the economic and industrial importance of this bioactive compound.

In traditional medicine, menthol and menthone-rich products and essential oils are used as repellents against insects to cure a variety of ailments, including some infections. The biological features of menthol—its analgesic, antibacterial, antifungal, anesthetic, and penetration-enhancing activities as well as chemopreventive and immunomodulating functions—have been studied in vitro and in vivo (22).

We analyzed the previously documented information about menthol in the ChEMBL 35 database (as described in materials and methods, Section 2.9). According to the literature review, antibacterial activity was found only for *Staphylococcus aureus*, where menthol reduced bacterial growth at high concentrations of 3.2 mM and 6.4 mM (23). In humans, menthol acted as a highly potent agonist of the bile acid receptor FXR (NR1H4), with an activity value of 2.2 nM. It displayed significant mutation-dependent modulation of TRPM8 ion channels. In human cells, menthol acted as a modulator with EC_50_ values ranging from 3 to 818 μM, influenced by specific mutations of the TRPM8 receptor (24,25,26).This monoterpenoid interfered with the interaction between the transcription factor RUNX1 and its binding partner CBF-beta, with a potency of 31.6 μM. Because RUNX1 is involved in blood cell development and certain cancers, this activity could be relevant to cancer biology. Menthol also showed activity against several disease-causing organisms. It inhibited *P*. *falciparum*, the parasite responsible for malaria, with a potency of 1.9 μM. Against SARS-CoV-2, menthol had an IC_50_ between 19.95 and 20 μM in Vero C1008 cells, suggesting moderate antiviral potential. It also caused mortality in *L*. *decemlineata* (0–46.7% mortality at 10 mg) (27) (28). However, menthol was much less effective against the plant fungus *Colletotrichum gloeosporioides*, with an EC_50_ of 2.9 mM (29), indicating low antifungal activity (Table S1). There is a large gap in our knowledge of antibacterial mechanisms of this monoterpenoid, despite the known fact of its antimicrobial influence.

Some authors mention the influence of menthol and menthol-bearing plants on membrane-associated properties of bacteria (17,30,31), but there is very little information available about the exact mechanisms of action of this substance.

The emergence and global spread of multidrug-resistant bacterial pathogens, including *Escherichia coli*, have intensified the search for novel antimicrobial agents and therapeutic strategies. In this context, natural plant-derived compounds, particularly menthol, have gained increasing attention due to their broad-spectrum antibacterial activity and potential to modulate microbial resistance mechanisms (13,32,33,34).

As it is well known, *E. coli* iofcapable of metabolic adaptations in response to oxygen availability. Under aerobic conditions, it relies on an oxidative phosphorylation process, ultimately generating ATP via the F_O_F_1_-ATP synthase complex. In contrast, under anaerobic or oxygen-limited conditions, the bacterium switches its metabolism to the fermentation pathways, during which ATP synthase can reverse its function to hydrolyze ATP, acting as an ATPase (35,36,37).

Many antimicrobial agents exert their effects through membrane disruption and disturbance of ion gradients, elucidating how menthol influences ATPase activity, ion fluxes (e.g., H^+^, K^+^), and bioenergetic properties of the cell under both aerobic and anaerobic conditions, which is critical to understand the mechanisms of its action. In addition, proton motive force, primarily driven by H^+^ translocation, is fundamental to bacterial ATP synthesis, nutrient uptake, and efflux pump function—a key mechanism contributing to antibiotic resistance in Gram-negative bacteria. The potassium ions also play an essential role in maintaining intracellular pH, membrane potential, and osmotic balance (38,39,40).

It has been suggested that plant-derived compounds may disrupt efflux pump function, thereby enhance intracellular accumulation of antibiotics and contribute to synergistic antibacterial effects. Therefore, the targeting of bacterial energy metabolism and ion homeostasis may represent a promising strategy for overcoming drug resistance in bacteria (41).

The present study aims to investigate the possible modes of suppressing action of menthol on antibiotic-resistant *E. coli* strains, with a specific focus on its impact on cell energetics. The experimental conditions were designed to assess bacterial responses under both aerobic and anaerobic environments, with and without N,N-dicyclohexylcarbodiimide (DCCD) inhibition, to provide comprehensive insights into the bioenergetic consequences of menthol exposure.

## Materials and Methods

### Bacterial strains and growth conditions

Two *E. coli* strains were applied in the experiments—*E. coli* BW 25113 (wild-type parent strain (Keio collection)) and *E. coli* pARG-25 (carrying pARG-25 plasmid (KanR) (high copy-cloning, kanamycin-resistant strain; the strain was kindly provided by the Microbial Depository Center at the Armbiotechnology Scientific and Production Center, Yerevan, Armenia.)). The strains were grown in liquid peptone medium (MP) of the following composition: 20 g L^-1^ peptone, 5 g L^-1^ NaCl, 2 g L^-1^ K_2_HPO_4_, and 2 g L^-1^ glucose, pH adjusted to 7.5 by 0.1 M NaOH or 0.1 M HCl. Bacteria were cultivated at 37°C, for 18–22 h in aerobic and anaerobic conditions. For aerobic growth, the cells were cultivated in rotary shaking conditions with 150 rpm (Multitron standart, Infors, Switzerland) (34).

### Investigation of antimicrobial activity

The antibacterial activity of menthol was initially determined by the b disk-diffusion and broth-dilution methods against the selected bacterial strains. The tests were performed using MP medium. The 2.5–1000 μg mL^-1^ concentration range of menthol was applied in the test. Ethanol (96%) was used as a negative control, and the antibiotic solutions (kanamycin (50 μg mL^-1^), ampicillin (50 μg mL^-1^)) were used as positive controls (42). Data were expressed as minimal inhibitory concentration (MIC) values.

### The growth kinetics of tested bacteria

The growth kinetics assay was employed to understand the pattern of bacterial growth under the influence of menthol. The growth kinetics of *E. coli* of both strains were monitored in the presence of menthol (12.5 and 125 μg mL^-1^). Fresh *E. coli* colonies were isolated from Mueller-Hinton agar plates and transferred to MP broth (pH 7.5) followed by incubation for 18 h at 37°C. Bacterial growth curves were determined by measuring the turbidity of samples containing bacteria at 565 ± 15 nm every 30 min with a densitometer (DEN-1B, BIOSAN, Latvia) (11).

Specific growth rate (μ) was calculated as the ratio of the logarithmic difference of doubled optical reading and doubling time when the bacterial growth curve was linear (11).

### Determination of ATPase activity in the membrane vesicles

Membrane vesicles of *E. coli* BW 25113 or *E. coli* pARG-25 bacteria were isolated according to the previously described method (43,44). Quantification of proteins was carried out by Lowry’s method, using Folin-Ciocalteu reagent (45). F_o_F_1_-ATPase activity in 100-μg membrane vesicles was determined by increasing inorganic phosphorus in the incubation medium (50 mM Tris-HCl buffer with 1 mM CaCl_2_ and 2.5 mM MgSvO_4_ (pH 7.5)) at 37°C according to the Tausski and Shorr method (46,47) using Cary 60 Agilent Technologies, Germany. For the determination of the DCCD-sensitive ATPase activity, the cells were incubated with 0.1 mM DCCD for 5–10 min, as described by Vanyan and Trchounian (48)(2022). The DCCD-sensitive ATPase activity was calculated as the difference between the samples with and without the inhibitor. Enzyme activity was expressed as nmol P_in_ min^-1^ mL^-1^ μg^-1^ protein.

### Determination of proton and potassium fluxes

Cells used for ion flux measurements were cultivated for 18–22 hours under both aerobic and anaerobic conditions and were harvested by centrifugation (Sorvall LYNX 6000 Superspeed Centrifuge, Thermo Scientific, USA) at 3500 × *g* for 15 min, and then the pellet was washed with distilled water twice. Pellet was resuspended in 150 mM Tris-HCl (pH 7.5) phosphate buffer containing 0.4 mM MgSO_4_, 1 mM NaCl, and 1 mM KCl. The proton (J_H+_) and potassium flux rates (J_K+_) were determined using potentiometric assay, by a pH/mV/ISE meter (HI5222, HANNA instruments, Portugal) equipped with a selective H^+^ electrode (HI1131) and potassium ion-selective electrode (HI-4114), by registering the pH and mV value changes, respectively, upon the addition of glucose (2 g L^-1^) and menthol (125 μg mL^-1^ and 12.5 μg mL^-1^) (48). J_H+_ was calculated as the negative logarithm of proton concentration in millimolar units. J_K+_ was calculated using the calibration curve of the electrode with different potassium concentrations and recording the mV for each concentration of potassium. For the determination of the DCCD-sensitive ion fluxes, the same principle was used as mentioned above. The results were expressed in mmol min^−1^ 10^8^ cells^−1^ in 1 unit of volume (mL).

### Determination of extracellular and intracellular pH

The extracellular pH (pH_ex_) was determined by using an ionometer (HI5222, HANNA instruments, Portugal) with pH-sensitive electrode (HI1131). The intracellular pH (pH_in_) was measured using 9-aminoacridine fluorescent dye (9-AA, with excitation at 339 nm and emission at 460 nm) with a Cary Eclipse, Spectrofluorimeter Agilent Technologies, Germany (48). Bacterial cells were harvested after 18–22 hours of growth by centrifugation (5430R centrifuge, Eppendorf, Germany). The harvested cells were washed with phosphate buffer (pH 6.5–7.0). 30 μM 9-AA, 20 μL of bacterial cell suspension (10^8^ cell mL^-1^), and 100 mM Tris-HCl buffers (pH 5.0, 5.5, 6.0, 6.5, 6.75, 7.0, 7.25, 7.5, 7.75, 8.0) were used for samples.

### Retrieval of bioactivity data for menthol from the ChEMBL database

Bioactivity data for menthol, focused on antibacterial action and influence on human cell lines, were collected using the SQLite version of the ChEMBL database (version 35) (49). Queries were performed directly on the local database to retrieve relevant entries, including compound-target interactions, assay details, and associated activity measurements. This approach made it easy to access well-organized bioactivity data related to menthol for further analysis.

In total, 118 activity reports related to menthol bioactivity were obtained. These entries were then processed: duplicates and records with incomplete data were removed. For targets with multiple reported activity types (such as IC_50_, EC_50_, or K_d_), the results were grouped by target and organism. For targets with multiple values reported under different experimental conditions, the data were consolidated, and the lowest and highest values were presented as a range to reflect the variability. After this refinement, the data set was reduced to 22 summarized activity reports, each representing a unique target or biological effect associated with menthol.

### Chemicals and reagents

Chemicals used were as follows: DL-Menthol (≥ 99%, synth., CAS No. 89-78-1, Carl Roth (Karlsruhe, Germany), ethanol (POCH S. A., lot no. 1156/11/21, Gliwice, Poland), kanamycin sulfate (#066M4019V, Sigma-Aldrich, Taufkirchen, Germany), tetracyclin (lot no. 60-54-8, Sigma-Aldrich, Taufkirchen, Germany), dimethyl sulfoxide (#BCCJ0028, Sigma-Aldrich, Taufkirchen, Germany), and DCCD (CAS No. 538−75−0, Sigma-Aldrich, Taufkirchen, Germany). Other applied chemicals and reagents were from Sigma-Aldrich (Taufkirchen, Germany) and VWR International (Pennsylvania, USA).

### Statistical analysis

All data presented represent averaged results of three independent biological replicates. The standard deviation of the data was determined according to the grouped two-way ANOVA test by using GraphPad Prism 8.0.3 data analysis tool and *p* < 0.05 (if not indicated). Graph presentations were carried out by GraphPad and Microsoft Excel 10 programs.

## Results

For the investigation of possible mechanisms of menthol action, two *E. coli* strains were selected—*E. coli* BW25113 and kanamycin-resistant *E. coli* pARG-25, as described in the materials and methods section. The *E. coli* BW25113 served as the wild-type strain. According to the initial testing assays the MIC value of menthol was documented to be 125 μg mL^-1^ for both *E. coli* BW25113 and kanamycin-resistant *E. coli* pARG-25 strains.

The MIC (125 μg mL^-1^) and the 10-time lower (12.5 μg mL^-1^) concentrations have been tested further. The application of the low concentration could be explained by the necessity of getting the bacterial biomass in order to be able to investigate the changes in bacterial metabolism.

**Figure 1:**
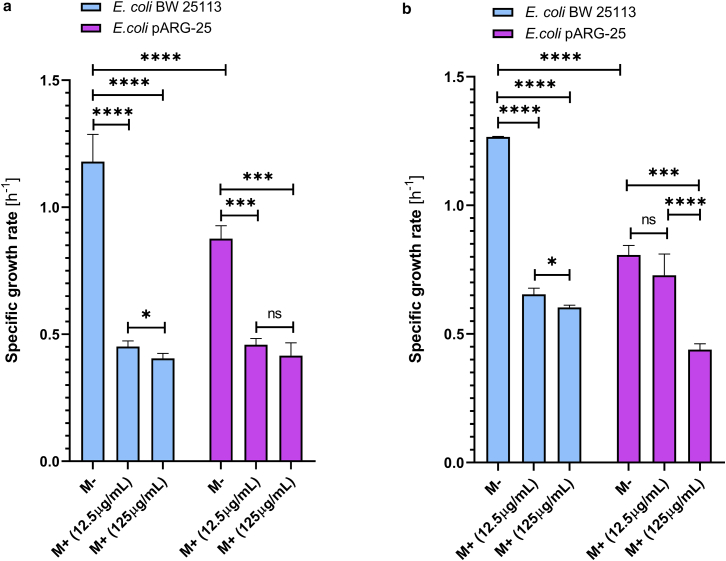
Impact of menthol on the specific growth rate of *E. coli* BW 25113 and kanamycin-resistant *E. coli* pARG-25 strains under the aerobic (*a*) and anaerobic (*b*) conditions. (M− are the control cells without treatment with menthol, and M+ are cells treated with menthol. The results are means ± SD of three independent experiments carried out in triplicate (ns, not significant; ∗∗∗*p* < 0.001; ∗∗∗∗*p* < 0.0001).

The investigations of the selected two *E. coli* strains showed that menthol expressed a suppressive effect on the growth of both strains in both aerobic and anaerobic conditions (Fig. 1, *a* and *b*).

In the case of the wild-type *E. coli* BW25113 strain, menthol suppressed the growth of the bacterium by 55% under aerobic and 40% under the anaerobic conditions compared with the control. The specific growth rate of the kanamycin-resistant strain was suppressed by 40% under the treatment with both menthol concentrations during aerobic growth conditions (the influence is not concentration dependent) (Fig. 1 *a*). Meanwhile, we observed the suppression differences between the selected two concentrations under the anaerobic conditions in case of the *E. coli* pARG-25 kanamycin-resistant strain (Fig. 1 *b*). The decreased concentration (12.5 μg mL^-1^) of menthol does not express any influence on the growth rate of kanamycin-resistant strain, whereas the selected higher concentration (which is the MIC) decreases the growth rate of bacteria by 40% (Fig. 1 *b*).

**Figure 2:**
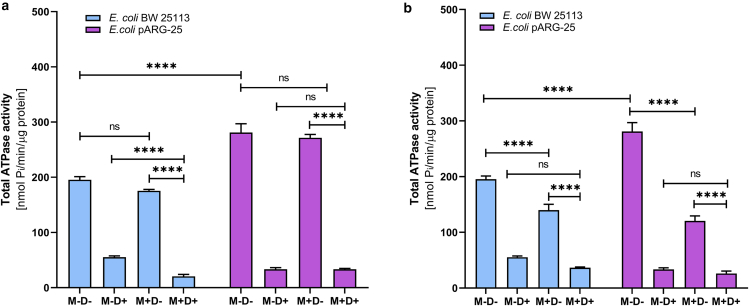
The impact of menthol on the total ATPase activity of *E. coli* BW 25113 and kanamycin-resistant *E. coli* pARG-25 membrane vesicles in aerobic conditions under the influence of 12.5 μg mL^-1^ (*a*) and 125 μg mL^-1^ (*b*) concentrations of menthol. M−D− are the control cells without the treatment with menthol and DCCD, M−D+ are the cells treated only with DCCD, M +D− are the cells treated only with menthol, and M+D+ are the cells treated with both menthol and DCCD. The results are means ± SD of three independent experiments carried out in triplicate (ns, not significant; ∗∗∗∗*p* < 0.0001).

We observed also that the total ATPase activity of the kanamycin-resistant *E. coli* pARG-25 strain was elevated under the initial conditions compared with the wild-type strain, which could be explained by the additional burden of carrying plasmids (Fig. 2).

Here, we observed a concentration-dependent influence of menthol on ATPase activity of both tested strains in both aerobic and anaerobic conditions. It was documented that the 12.5 μg mL^-1^ concentration of menthol does not express any significant influence on total ATPase activity of *E. coli* pARG-25 strain under the aerobic conditions (Fig. 2 *a*). Despite this, the MIC of menthol decreased the total ATPase activity twice that of the same strain (Fig. 2 *b*). In case of the *E. coli* BW 25113 strain the menthol MIC reduces this parameter by almost 40% (Fig. 2, *a* and *b*).

**Figure 3:**
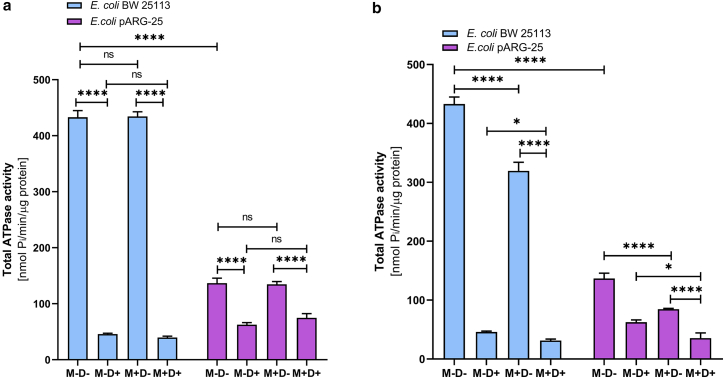
The impact of menthol on total ATPase activity of *E. coli* BW 25113 and kanamycin-resistant *E. coli* pARG-25 membrane vesicles in anaerobic conditions ((*a*) 12.5 μg mL^-1^ and (*b*) 125 μg mL^-1^ concentrations of menthol). M−D− are control cells without treatment with menthol and DCCD, M−D+ are cells treated only with DCCD, M+D− are cells treated only with menthol, and M+D+ are the cells treated with both menthol and DCCD. The results are means ± SD of three independent experiments carried out in triplicate (ns, not significant; ∗*p* < 0.05; ∗∗∗∗*p* < 0.0001).

The *E. coli* BW 25113 strain expresses higher total ATPase activity compared with the kanamycin-resistant strain under the anaerobic conditions (Fig. 3 *a*). Again, in case of the wild-type strain, the 12.5μg mL^-1^ concentration of menthol does not express any influence on bacteria, although the higher concentration decreases the total ATPase activity by around 35% (Fig. 3, *a* and *b*).

The decreasing of the total ATPase activity in the kanamycin-resistant strain reached 60%, in the case of the treatment of cells with 125 μg mL^-1^ concentration of menthol under the anaerobic conditions, whereas the lower tested concentration of menthol did not express any influence (Fig. 3, *a* and *b*).

**Figure 4:**
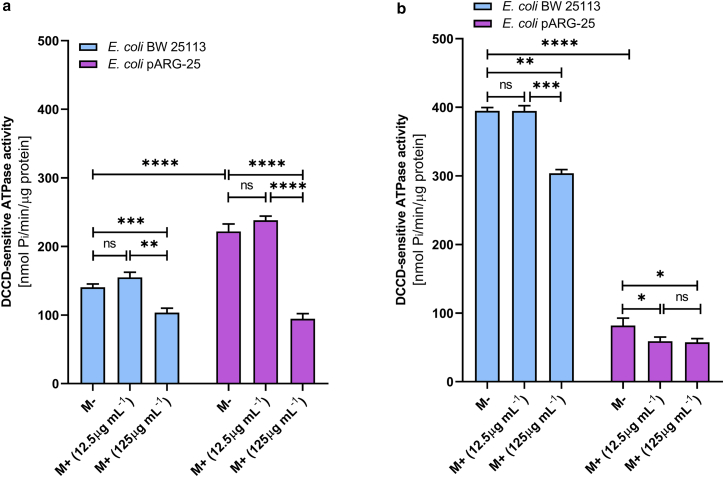
The impact of menthol on DCCD-sensitive ATPase activity of *E. coli* BW 25113 and *E. coli* pARG-25 membrane vesicles in aerobic (*a*) and anaerobic (*b*) conditions with 12.5 μg mL^-1^ and 125 μg mL^-1^ concentrations of menthol. M−D− are the control cells without treatment with menthol and DCCD, M−D+ are the cells treated only with DCCD, M+D− are the cells treated only with menthol, and M+D+ are the cells treated with both menthol and DCCD. The results are means ± SD of three independent experiments, each carried out in triplicate (ns, not significant; ∗*p* < 0.05; ∗∗*p* < 0.01; ∗∗∗*p* < 0.001; ∗∗∗∗*p* < 0.0001).

In order to investigate the effect of menthol on proton-translocating ATPase activity, the DCCD-sensitive ATPase was investigated in membrane vesicles isolated from both *E. coli* BW25113 and *E. coli* pARG-25 strains under aerobic and anaerobic conditions after the treatment with menthol (12.5 μg/mL and 125 μg/mL) (Fig. 4).

Under aerobic conditions (Fig. 4 *a*), the DCCD-sensitive ATPase activity (M−D−) in *E. coli* pARG-25 was significantly higher than in the *E. coli* BW25113 control strain (*p* < 0.0001). The treatment with menthol led to a concentration-dependent decrease in ATPase activity in both strains. In *E. coli* BW25113 strain, significant inhibition was observed only at the higher menthol concentration (125 μg mL^-1^; *p* < 0.001), whereas in *E. coli* pARG-25 cells, both concentrations of menthol caused a significant reduction in activity (*p* < 0.0001). Notably, ATPase activity remained elevated in *E. coli* pARG-25 compared with *E. coli* BW25113 across all conditions, except the presence of menthol MIC.

During anaerobic conditions (Fig. 4 *b*), the DCCD-sensitive ATPase activity in *E. coli* BW25113 was markedly higher than the same parameter documented for the kanamycin-resistant *E. coli* pARG-25 strain (*p* < 0.0001). In *E. coli* BW25113, menthol only at 125 μg mL^-1^ concentration significantly reduced ATPase activity (*p* < 0.0001). In contrast, ATPase activity in *E. coli* pARG-25 strain cells was substantially lower and exhibited a modest but statistically significant decrease upon the treatment with 125 μg mL^-1^ of menthol (*p* < 0.01), though no significant difference was observed between the two menthol concentrations (*p* > 0.05).

**Figure 5:**
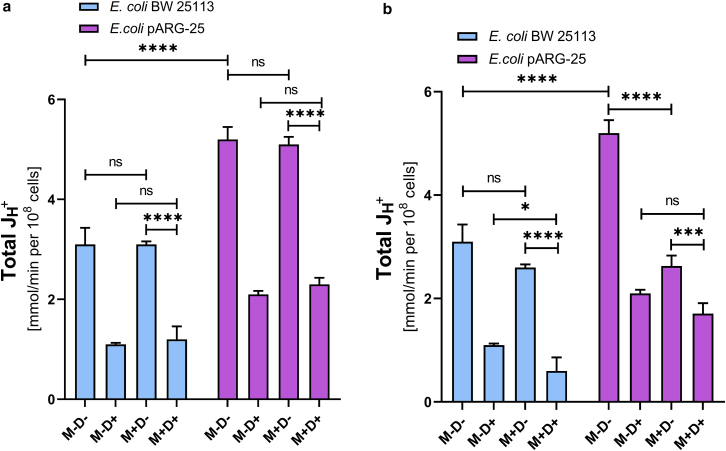
The effect of menthol on total H^+^-fluxes through the *E. coli* BW 25113 and kanamycin-resistant *E. coli* pARG-25 membranes under the aerobic conditions (under the treatment with 12.5 μg mL^-1^ (*a*) and 125 μg mL^-1^ (*b*) concentrations of menthol). M−D− are the control cells without the treatment with menthol and DCCD, M−D+ are the cells treated only with DCCD, M+D− are the cells treated only with menthol, and M+D+ are the cells treated with both menthol and DCCD. The results are means ± SD of two independent experiments carried out in triplicate (ns, not significant; ∗*p* < 0.05; ∗∗∗*p* < 0.001; ∗∗∗∗*p* < 0.0001).

As anticipated, the total proton flux is higher in kanamycin-resistant *E. coli* pARG-25 strain compared with the wild-type *E. coli* under aerobic conditions (Figs. 2 and 5). Exposure to 12.5 μg mL^-1^ menthol does not significantly alter the total proton flux in either strain. However, treatment with menthol at its minimum inhibitory concentration (MIC) results in a 1.2-fold reduction in proton flux in the wild-type strain and a twofold decrease in the kanamycin-resistant *E. coli* pARG-25 strain (Fig. 5, *a* and *b*).

**Figure 6:**
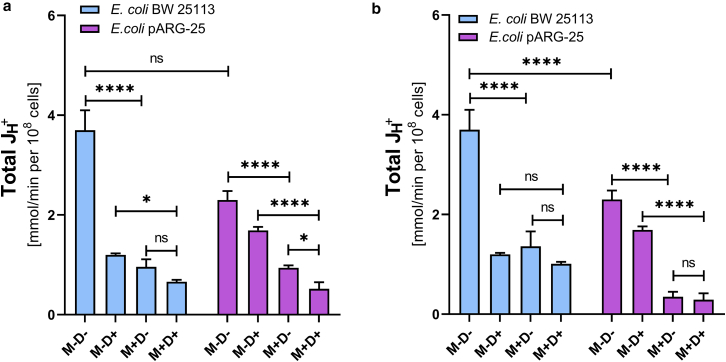
The effect of menthol on total H^+^-fluxes through the *E. coli* BW 25113 and kanamycin-resistant *E. coli* pARG-25 membranes in anaerobic conditions (under the treatment with 12.5 μg mL^-1^ (*a*) and 125 μg mL^-1^ (*b*) concentrations of menthol). M−D− are the control cells without the treatment with menthol and DCCD, M−D+ are the cells treated only with DCCD, M+D− are the cells treated only with menthol, and M+D+ are the cells treated with both menthol and DCCD. The results are means ± SD of two independent experiments carried out in triplicate (ns, not significant; ∗*p* < 0.05; ∗∗∗*p* < 0.001; ∗∗∗∗*p* < 0.0001).

During anaerobic conditions, similar to the pattern observed for total ATPase activity, the total proton flux in untreated samples is higher in the wild-type *E. coli* BW25113 strain compared with the kanamycin-resistant *E. coli* pARG-25 strain across both tested concentrations of menthol (Fig. 6).

In the wild-type strain, menthol at 12.5 μg mL^-1^ concentration reduces proton flux by ∼30%, whereas treatment with the menthol at MIC results ∼40% reduction. In contrast, the kanamycin-resistant strain exhibits a more pronounced concentration-dependent response: proton flux decreased ∼60% and ∼80% at the at 12.5 μg mL^-1^ and MIC, respectively.

In the presence of menthol, no significant difference was observed in total proton flux before and after the treatment with DCCD in either strain across both tested menthol concentrations (Fig. 6, *a* and b). In the wild-type strain, menthol does not significantly affect the DCCD-sensitive component of proton flux under aerobic conditions.

**Figure 7:**
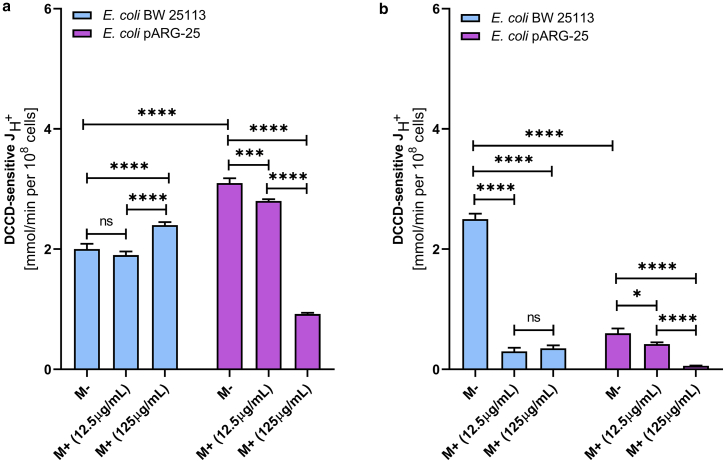
The impact of menthol on DCCD-sensitive H^+^-fluxes through the *E. coli* BW 25113 and *E. coli* pARG-25 membranes under the aerobic (*a*) and anaerobic (*b*) conditions and the treatment with 12.5 μg mL^-1^ and 125 μg mL^-1^ concentrations of menthol. M−D− are the control cells without treatment with menthol and DCCD, M−D+ are the cells treated only with DCCD, M+D− are the cells treated only with menthol, and M+D+ are the cells treated with both menthol and DCCD. The results are means ± SD of three independent experiments, each carried out in triplicate (ns, not significant; ∗*p* < 0.05; ∗∗∗*p* < 0.001; ∗∗∗∗*p* < 0.0001).

In contrast, under anaerobic conditions, the treatment with menthol leads to an 90% inhibition of DCCD-sensitive flux, irrespective of its concentration (Fig. 7 *b*). In case of the kanamycin-resistant *E. coli* strain, the treatment with menthol at MIC reduces the DCCD-sensitive proton flux by approximately 70% under aerobic and by 80% under anaerobic conditions (Fig. 7, *a* and b).

**Figure 8:**
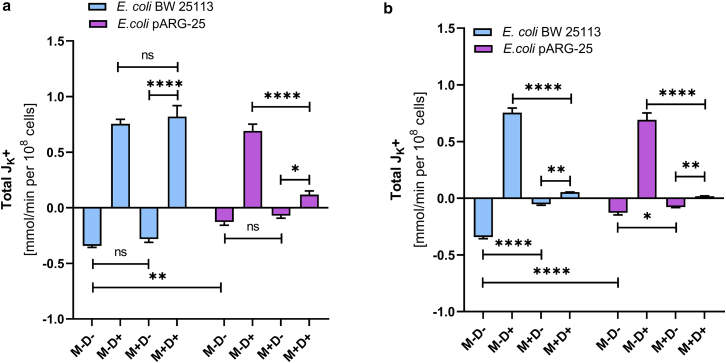
The effect of menthol on total K^+^-fluxes through the *E. coli* BW 25113 and kanamycin-resistant *E. coli* pARG-25 membranes in aerobic conditions with 12.5 μg mL^-1^ (*a*) and 125 μg mL^-1^ (*b*) concentration of menthol. M−D− are the control cells without treatment with menthol and DCCD, M−D+ are the cells treated only with DCCD, M+D− are the cells treated only with menthol, and M+D+ are the cells treated with both menthol and DCCD. The results are means ± SD of two independent experiments carried out in triplicate (ns, not significant; ∗*p* < 0.05; ∗∗*p* < 0.01; ∗∗∗*p* < 0.001; ∗∗∗∗*p* < 0.0001).

The exposure of 12.5 μg mL^-1^ menthol does not significantly affect total K^+^ flux in either strain under aerobic conditions (Fig. 8 *a*). However, at the MIC, potassium flux is reduced by 85% in the wild-type strain and by 40% in the kanamycin-resistant strain (Fig. 8 *b*). Although low menthol concentration has minimal effect on K^+^ flux in *E. coli* BW25113, the treatment with 125 μg mL^-1^ concentration of menthol suppresses K^+^ flux by approximately 80%. In the antibiotic-resistant strain, potassium flux is reduced by 45% (in case of applying the 12.5 μg mL^-1^ concentration) and by 40% (in case of 125 μg mL^-1^ concentration of menthol) (Fig. 8).

**Figure 9:**
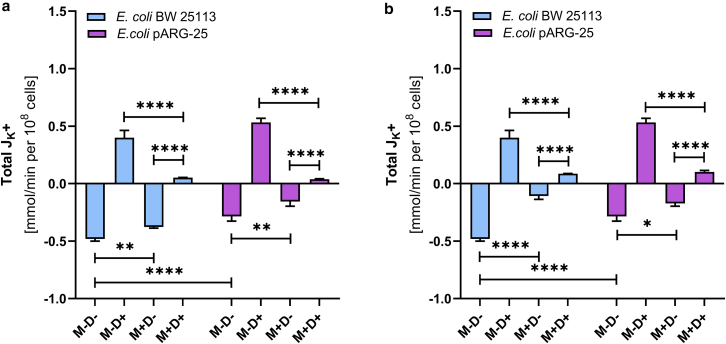
The effect of menthol (12.5 μg mL^-1^ (*a*) and 125 μg mL^-1^ (*b*)) on total K^+^-fluxes through the *E. coli* BW 25113 and kanamycin-resistant *E. coli* pARG-25 membranes in anaerobic conditions. M−D− are the control cells without the treatment with menthol and DCCD, M−D+ are the cells treated only with DCCD, M+D− are the cells treated only with menthol, and M+D+ are the cells treated with both menthol and DCCD. The results are means ± SD of two independent experiments carried out in triplicate (∗*p* < 0.05; ∗∗*p* < 0.01; ∗∗∗∗*p* < 0.0001).

Menthol, at its MIC, significantly inhibits the DCCD-sensitive potassium flux in both strains under aerobic conditions. In the wild-type *E. coli* BW25113 strain, the higher concentration of menthol (125 μg mL^-1^) reduced the DCCD-sensitive K^+^ flux by approximately 90%, whereas in the kanamycin-resistant *E. coli* pARG-25 strain, the reduction was about 50% (Fig. 9).

Menthol influences also the intracellular pH of both tested strains (Table 1). In aerobic conditions the ΔpH value was twice lower in the wild-type strain under the treatment of menthol MIC, meanwhile in case of antibiotic-resistant *E. coli*, this parameter value was around five times lower under the treatment of menthol, which could speak about the disruption in bacterial pH homeostasis. The same tendency was observed also in anaerobic conditions (Table 1).

**Table 1:** The effect of menthol on intracellular and extracellular pH of the *E. coli* BW 25113 and kanamycin-resistant *E. coli* pARG-25 strains treated with 125 μg mL^-1^ concentration of menthol

	Aerobic				Anaerobic			
	*E. coli* BW25113		*E. coli* pARG-25		*E. coli* BW25113		*E. coli* pARG-25	
	M−	M+ (125 μg mL^-1^)	M−	M+ (125 μg mL^-1^)	M−	M+ (125 μg mL^-1^)	M−	M+ (125 μg mL^-1^)
Intracellular pH	7.0	6.75	7.1	6.6	6.9	6.6	6.6	6.4
Extracellular pH	6.6	6.55	6.67	6.51	6.66	6.49	6.51	6.38
ΔpH	−0.4	−0.2	−0.43	−0.09	−0.24	−0.11	−0.1	−0.02

**Figure 10:**
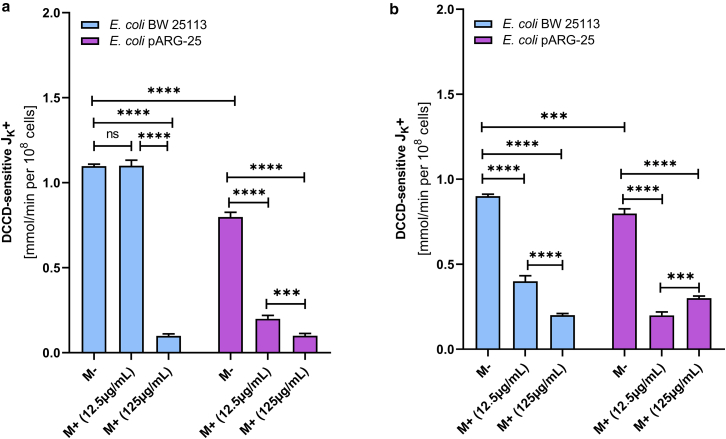
The impact of menthol (12.5 μg mL^-1^ and 125 μg mL^-1^) on DCCD-sensitive K^+^-fluxes through the *E. coli* BW 25113 and *E. coli* pARG-25 membranes in aerobic (*a*) and anaerobic (*b*) conditions. M−D− are the control cells without treatment with menthol and DCCD, M−D+ are the cells treated only with DCCD, M+D− are the cells treated only with menthol, and M+D+ are the cells treated with both menthol and DCCD. The results are means ± SD of three independent experiments, each carried out in triplicate (∗∗∗*p* < 0.001; ∗∗∗∗*p* < 0.0001).

A similar inhibitory pattern is observed under anaerobic conditions. In the wild-type strain, DCCD-sensitive potassium flux is decreased by 50% and 80% after the treatment with 12.5 μg mL^-1^ and 125 μg mL^-1^ concentrations of menthol, respectively. In the antibiotic-resistant *E. coli* strain, both concentrations of menthol also reduce DCCD-sensitive K^+^ flux under anaerobic conditions, although to a lesser extent (Fig. 10; Table 2).

**Table 2:** DCCD-sensitive proton/potassium flux ratio

*E. coli* BW25113			*E. coli* pARG-25		
M−	M+ (12.5 μg mL^-1^)	M+ (125 μg mL^-1^)	M−	M+ (12.5 μg mL^-1^)	M+ (125 μg mL^-1^)
Aerobic conditions					
1.8	1.1	22.4	3.8	14.8	9.4
Anaerobic conditions					
2.8	0.7	2.1	0.7	2.1	0.4

## Discussion

Our previous data of *M. arvensis* essential oil demonstrated that monoterpenes represent the predominant chemical class in its composition, with menthol identified as the principal component, reaching approximately 69.75% of the total content. Over 30 additional constituents were detected in minor amounts (50). Given the high abundance of menthol and the observed antibacterial activity of *M. arvensis* essential oil against both Gram-positive and Gram-negative bacteria, we aimed to determine whether menthol alone was responsible for the bioactivity or if synergistic effects from minor components also contributed. As it was determined, menthol inhibited the growth of the kanamycin-resistant *E. coli* pARG-25 strain. It is known that kanamycin disrupts protein synthesis in bacteria by binding to the 30S ribosomal subunit, requiring active transport of acting substance across the membrane. These findings suggested a possible membrane-related mechanisms of menthol action (51).

We have shown that the MIC of menthol against *E. coli* is 125 μg mL^-1^, which corresponds to approximately 800 μM. This MIC value is stronger than those observed for Gram-positive *Staphylococcus aureus*, where menthol reduced bacterial growth at 6.4 mM and 3.2 mM (23) and fungal pathogens, where activity was seen at higher concentrations (Table S1). Therefore, our results indicate that menthol is more effective against Gram-negative *E. coli*.

Although previous studies have noted the membrane-disruptive potential of menthol (17,19,52,53), detailed insights into its biochemical mechanisms on membrane-bound enzymes and cell bioenergetic properties remain limited. To address this gap, we investigated the impact of menthol on ion fluxes (H^+^ and K^+^) and FoF_1_-ATPase activity under aerobic and anaerobic growth conditions in the wild-type *E. coli* BW25113 and kanamycin-resistant *E. coli* pARG-25 strains.

The aerobic conditions contributed to the concentration-dependent inhibitory action of menthol on ATPase activity, with more pronounced effects in the kanamycin-resistant strain. This elevated activity may be attributed to the presence of the plasmid carrying the antibiotic resistance genes, which imposes an additional energetic burden on the cells due to plasmid replication and the expression of resistance genes, thereby increasing the demand for ATP to maintain normal cellular functions (54). Besides, it was known that when the cell is under different stress conditions, it tries to survive with regulating the F_o_F_1_-ATPase activity (55,56).

In *E. coli* BW25113 wild-type cells, 12.5 μg/mL menthol did not significantly affect total or DCCD-sensitive proton flux, which is in accordance with unaltered ATPase activity. However, the specific growth rate was reduced, indicating alternative mechanisms of action, potentially not related to direct interference with proton transport. At 125 μg/mL concentration, menthol inhibited ATPase activity and enhanced DCCD-sensitive proton flux, implicating the FoF_1_-ATPase as a potential target in the wild-type strain. In *E. coli* pARG-25 cells, ATPase sensitivity to DCCD decreased under the treatment of the menthol MIC, suggesting that menthol may occupy or modify the binding site of DCCD, preventing inhibition by DCCD. This idea needs further clarification and deeper experimental evidence. But the idea suggested is one of the interpretations that can cause this effect. DCCD insensitivity of F_O_F_1_ was shown for Mediterranean mussel mitochondria due to the presence of lipophilic pollutant tributyltin (57), which might affect the structural changes in the F_O_ part.

Under anaerobic fermentative conditions, menthol had a strong inhibitory effect on proton flux in kanamycin-resistant *E. coli* strain in a concentration-dependent manner. At 125 μg/mL, total proton flux was suppressed about sixfold, and DCCD-sensitive activity was abolished. This supports the hypothesis that menthol might directly interfere with FoF_1_-ATPase or alternatively change the structure of F_O_, altering the activity or possibly preventing DCCD binding and blocking proton extrusion.

In addition to proton fluxes, menthol also altered potassium ion fluxes. Under aerobic conditions in *E. coli* BW25113 wild-type cells, 12.5 μg/mL menthol induced potassium efflux via a DCCD-sensitive mechanism, suggesting involvement of Trk or other systems. At 125 μg/mL, potassium flux was reduced, likely due to suppressed ATPase activity and overall membrane disruption. Interestingly, under anaerobic fermentative conditions, low concentration of menthol had negligible effects, whereas 125 μg/mL impacted both ATPase activity and potassium flux. This might be explained by the metabolic differences during aerobic and anaerobic conditions.

In contrast, *E. coli* pARG-25 displayed increased tolerance to menthol, particularly under aerobic conditions, where Trk system involvement appeared diminished. However, under anaerobic conditions, 125 μg/mL menthol inhibited the specific growth rate, ATPase activity, and potassium flux by 40%, reinforcing its membrane-targeting potential. It is important to note that in potassium flux measurements in wild-type and mutant strain in both aerobic and anaerobic conditions, addition of DCCD resulted in the potassium efflux from the cell, which might be explained due to the decreased membrane potential, whereas in menthol assays with DCCD, no such effects were determined. These data once more suggest possible direct influence of menthol on F_O_F_1_ resulting in different actions on potassium fluxes. Under the anaerobic conditions, other systems of ion gradient regulations might play.

Menthol also influences the intracellular pH of both tested strains (Table 1). In aerobic conditions the ΔpH value was twice lower in wild-type strain under the treatment of menthol MIC; meanwhile, in the case of antibiotic-resistant *E. coli*, it was around fivefold lower in the presence of menthol (Table 1), which might tell about the alteration in bacterial pH homeostasis. The same tendency was observed also in anaerobic conditions (Table 1). As we know, menthol decreased F_0_F_1_-ATPase activity and total potassium flux, suggesting the role of menthol on the modulation of membrane potential, as both the F_0_F_1_-ATPase complex and K^+^ flux are key contributors to its generation (58). Menthol enhances the influx of DCCD-sensitive potassium ions under both aerobic and anaerobic conditions, affecting the ΔpH.

**Figure 11:**
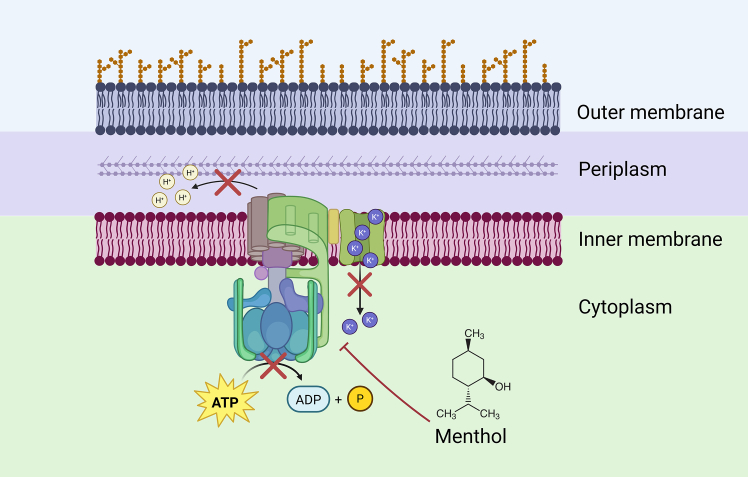
Schematic representation of inhibitory effects of menthol on proton and potassium fluxes in *E. coli* during anaerobic fermentation. Menthol disrupts F_o_F_1_-ATPase activity and alters DCCD-sensitive (F_O_F_1_ dependent) ion (proton and potassium) fluxes.

Taken together, our findings suggest that menthol exerts its antibacterial effects through multiple mechanisms, prominently including the disruption of key bioenergetic parameters, particularly altering the activity of FoF_1_-ATPase, modulating ion fluxes, and disturbing intracellular pH (59). Particularly, it might be suggested that menthol affects the F_O_ part of the proton ATPase and thus unravels the F_O_F_1_-dependent ion balance. These effects are concentration, oxygen, and strain dependent, with more pronounced impacts observed in the kanamycin-resistant *E. coli* strain during anaerobic fermentative conditions (Fig. 11). These insights contribute to a better understanding of the antibacterial mechanisms of menthol and its potential as an agent against antibiotic-resistant bacteria.

In comparison to previous reports, our results align with earlier findings that highlight the antimicrobial activity of menthol against both Gram-positive and Gram-negative bacteria. For example, Freires et al. (2015) emphasized menthol’s membrane-disrupting action, which leads to leakage of intracellular contents (60). Sahoo et al. (2022) also reported moderate antibacterial activity of menthol and eucalyptus oil against *E. coli* and *S. aureus* (61). Turcheniuk et al. (2015) demonstrated that menthol, when conjugated with nanodiamonds, significantly inhibited biofilm formation in *E. coli*, though the conjugated form was less bactericidal than free menthol, likely due to reduced membrane interaction (62). Notably, Schelz et al. (2006) reported a much higher MIC of 525 μg/mL for *E. coli* (63), whereas our study identified a significantly lower MIC of 125 μg mL^-1^. This discrepancy might be attributed to differences in bacterial strains, methodologies, or test conditions. In contrast, Aperce et al. (2016) found no reduction in *E. coli* counts in cattle following menthol dietary supplementation, suggesting that systemic administration or complex matrices may limit its antimicrobial efficacy (64). Overall, our findings provide mechanistic insights and highlight the importance of direct cellular interaction in menthol’s antibacterial effect.

In addition, it could also be suggested that menthol might be able to insert into the membrane due to its lipophilic nature and cause some disruption and malfunction such as alterations in ion gradients, particularly those of the ATPase and H^+^/K^+^ transporting systems and pH homeostasis, which try to compensate for the membrane defects by adjusting their activities (65). This partly explains why the effects in the antibiotic-resistant *E. coli* strain are stronger than in the nonresistant one.

Our findings not only deepen our understanding of antibiotic resistance mechanisms but also hold promise for the development of novel treatment strategies to combat this pressing global health challenge. Although, further research is required to elucidate the specific mechanisms through which menthol exerts its antibacterial effects. Understanding these mechanisms could pave the way for the development of new antimicrobial agents and contribute to combating antibiotic resistance in pathogenic bacteria.

## Acknowledgments

We are thankful to Dr. Anahit Shirvanyan for her contribution and support in some measurements. The research was supported by the Higher Education and Science Committee of MESCS RA, in the frames of the research projects № 24WS-1F003.

## Author contributions

All authors contributed to the study’s conception and design. S.T. carried out the investigations and analyzed data, S.G., H.G., and R.A. were responsible for the bioinformatics, and K.T. and N.A. analyzed the outcomes and wrote the manuscript. K.T., R.A., and N.S. directed the experiments and corrected and edited the manuscript. All authors revised and accepted the final version of the manuscript.

## Declaration of interests

The authors declare no competing interests.

## Supporting Material

Document S1. Table S1

Document S2. Article plus Supporting Material
