# Supplementary material for: Menthol’s disruptive effects on kanamycin-resistant Escherichia coli energy metabolism and ion fluxes
Source: Biophys Rep (N Y). 2025 Nov 20;5(4):100240. doi: 10.1016/j.bpr.2025.100240 (PMC12720088; doi:10.1016/j.bpr.2025.100240)
Supplement: Document S1. Table S1 [file mmc1.pdf]

Biophysical Reports, Volume *5*

Supplemental information

Menthol's disruptive effects on kanamycin-resistant *Escherichia coli* energy metabolism and ion fluxes

Silvard Tadevosyan, Siranuysh Grabska, Hovakim Grabski, Ruben Abagyan, Karen Trchounian, and Naira Sahakyan

Menthol Disruptive Effects on Kanamycin-Resistant Escherichia coli Energy Metabolism and Ion Fluxes

Silvard Tadevosyan[1], Siranuysh Grabska[2], Hovakim Grabski[2], Ruben Abagyan[3], Karen Trchounian[1]* and Naira Sahakyan[1]**

[1]Research Institute of Biology, Yerevan State University Department of Biochemistry, Microbiology & Biotechnology, Yerevan State University, Yerevan, Armenia

[2]L.A. Orbeli Institute of Physiology, National Academy of Sciences, Yerevan, Armenia

[3] Skaggs School of Pharmacy and Pharmaceutical Sciences, University of California, La Jolla, San Diego, CA 92093-0657, USA

***Correspondence:** k.trchounian@ysu.am; sahakyannaira@ysu.am

Supporting table 1. Bioactivity profile of menthol against various biological targets.

Target	Gene names	Mutation	Target organism	Activity*	Activity type	DOI or Chembl assay ID	Assay cell type
Bile acid receptor FXR	NR1H4, BAR, FXR, HRR1, RIP14	-	*Homo sapiens*	2.2 nM	Agonist	CHEMBL 1794415	-
Colletotrichum gloeosporioides	-	-	*Colletotrichum gloeosporioides*	EC50=2.9 mM	Inhibition	(26)	-
Plasmodium falciparum	-	-	*Plasmodium falciparum*	1.9 µM	Inhibition	CHEMBL 1794345	-
Runt-related transcription factor 1/Core-binding factor subunit beta	RUNX1, AML1, CBFA2	-	*Homo sapiens*	31.6 µM	Blocking the Interaction Between CBF-beta and RUNX1	CHEMBL 1613933	-
SARS-CoV-2	-	-	*Severe acute respiratory syndrome coronavirus*	*IC50= [19.95, 20] µM	Inhibition	(27)	Vero C1008
Staphylococcus aureus strain MN8	-	-	*Staphylococcus aureus*	6.4 mM<br>3.2 mM	% Control (Growth):<br>32%<br>119%	(25)	-

Staphylococcus aureus strain MN8	-	-	*Staphylococcus aureus*	6.4 mM 3.2 mM	% Control (toxic shock syndrome toxin 1 (TSST-1)): 1.8% 24.4%	(25)	-
GABA receptor			*Musca domestica*	500 µM	80% inhibition of [3H]-TBOB binding	(29)	-
Leptinotarsa decemlineata	-	-	*Leptinotarsa decemlineata*	10 mg 20 mg	Mortality (Exposure time: 12-96 h): 0-46.7% 0-40%	(30)	
Transient receptor potential cation channel subfamily A member 1	TRPM1, LTRPC, MLSN, MLSN1	-	*Homo sapiens*	EC50=28.4 µM *EC50 [30, 31.6] µM	Activator	(31, 32)	HEK293
Transient receptor potential cation channel subfamily A member 1	TRPM1, LTRPC, MLSN, MLSN1	-	*Mus musculus*	IC50=56µM EC50=95µM	Modulator (Activator or Inhibitor)	(33)	CHO
Transient receptor potential cation channel subfamily M member 8	TRPM8, LTRPC, TRPP8	-	*Homo sapiens*	10 µM *EC50 [3, 29] µM IC50=92µM Kd=14.4µM	Modulator (Activator or Inhibitor)	(34–36)	HEK293

Transient receptor potential cation channel subfamily M member 8	TRPM8, LTRPC, TRPP8	K856A	*Homo sapiens*	EC50=11.7 µM IC50=19µM	Modulator (Activator or Inhibitor)	(35)	HEK293
Transient receptor potential cation channel subfamily M member 8	TRPM8, LTRPC, TRPP8	H845A	*Homo sapiens*	EC50=34.1 µM	Modulator (Activator or Inhibitor)	(35)	HEK293
Transient receptor potential cation channel subfamily M member 8	TRPM8, LTRPC, TRPP8	K856R	*Homo sapiens*	EC50=35.3 µM	Modulator (Activator or Inhibitor)	(35)	HEK293
Transient receptor potential cation channel subfamily M member 8	TRPM8, LTRPC, TRPP8	R851Q	*Homo sapiens*	EC50=38.4 µM	Modulator (Activator or Inhibitor)	(35)	HEK293
Transient receptor potential cation channel subfamily M member 8	TRPM8, LTRPC, TRPP8	R862A	*Homo sapiens*	EC50=38.8 µM	Modulator (Activator or Inhibitor)	(35)	HEK293
Transient receptor potential cation channel subfamily M member 8	TRPM8, LTRPC, TRPP8	-	*Rattus norvegicus*	EC50=75µM	-	(37)	HEK293

Transient receptor potential cation channel subfamily M member 8	TRPM8, LTRPC6, TRPP8	R842K	*Homo sapiens*	EC50=93µM	Modulator (Activator or Inhibitor)	(35)	HEK293
Transient receptor potential cation channel subfamily M member 8	TRPM8, LTRPC6, TRPP8	R842A	*Homo sapiens*	EC50=818 µM IC50=520 µM	Modulator (Activator or Inhibitor)	(35)	HEK293
Vitamin D receptor	VDR, NR1I1	-	*Homo sapiens*	39.8 µM	Agonist	CHEMBL 1794311	-

* Activity values across tested conditions.
